# Enhancing Polyvinyl Alcohol Nanocomposites with Carboxy-Functionalized Graphene: An In-Depth Analysis of Mechanical, Barrier, Electrical, Antibacterial, and Chemical Properties

**DOI:** 10.3390/polym16081070

**Published:** 2024-04-11

**Authors:** Yu-Jie Shui, Wei-Hua Yao, Jarrn-Horng Lin, Yingjun Zhang, Yongqi Yu, Chin-San Wu, Xuemei Zhang, Chi-Hui Tsou

**Affiliations:** 1 Material Corrosion and Protection Key Laboratory of Sichuan Province, School of Materials Science and Engineering, Sichuan University of Science and Engineering, Zigong 643000, China; 2 Department of Materials and Textiles, Asia Eastern University of Science and Technology, New Taipei City 220, Taiwan; 3 Department of Material Science, National University of Tainan, Tainan 70005, Taiwan; 4 Department of Applied Cosmetology, Kao Yuan University, Kaohsiung 82101, Taiwan

**Keywords:** polyvinyl alcohol, carboxy-functionalized graphene, nanocomposite films, hydrophobicity, barrier performance, antibacterial, conductivity

## Abstract

To enhance the various properties of polyvinyl alcohol (PVA), varying concentrations of carboxy-functionalized graphene (CFG) were employed in the preparation of CFG/PVA nanocomposite films. FTIR and XRD analyses revealed that CFG, in contrast to graphene, not only possesses carboxylic acid group but also exhibits higher crystallinity. Mechanical testing indicated a notable superiority of CFG addition over graphene, with optimal mechanical properties such as tensile and yield strengths being achieved at a 3% CFG concentration. Relative to pure PVA, the tensile strength and yield strength of the composite increased by 2.07 and 2.01 times, respectively. XRD analysis showed distinct changes in the crystalline structure of PVA with the addition of CFG, highlighting the influence of CFG on the composite structure. FTIR and XPS analyses confirmed the formation of ester bonds between CFG and PVA, enhancing the overall performance of the material. TGA results also demonstrated that the presence of CFG enhanced the thermal stability of CFG/PVA nanocomposite films. However, analyses using scanning electron microscopy and transmission electron microscopy revealed that a 3% concentration of CFG was uniformly dispersed, whereas a 6% concentration of CFG caused aggregation of the nanofiller, leading to a decrease in performance. The incorporation of CFG significantly enhanced the water vapor and oxygen barrier properties of PVA, with the best performance observed at a 3% CFG concentration. Beyond this concentration, barrier properties were diminished owing to CFG aggregation. The study further demonstrated an increase in electrical conductivity and hydrophobicity of the nanocomposites with the addition of CFG. Antibacterial tests against *E. coli* showed that CFG/PVA nanocomposites exhibited excellent antibacterial properties, especially at higher CFG concentrations. These findings indicate that CFG/PVA nanocomposites, with an optimized CFG concentration, have significant potential for applications requiring enhanced mechanical strength, barrier properties, and antibacterial capabilities.

## 1. Introduction

The realm of polymer materials, which are pivotal to a vast array of industrial applications, often grapples with inherent limitations such as inadequate mechanical strength, subpar oxygen resistance, and compromised water resistance. These shortcomings significantly hamper the utility of polymer materials in demanding environments, necessitating the development of advanced materials that can overcome these challenges. In this context, polymer composites have emerged as a beacon of innovation, offering enhanced properties and broadening the application spectrum of base polymers [1]. The genesis of polymer nanocomposites is rooted in the infusion of nanoparticles or additives into polymer matrices. This strategy has been extensively explored, leading to the consideration of diverse fillers such as carbon nanotubes (CNTs) [2,3,4], graphene [5,6,7], fullerenes [8], carbon fibers [9], various metals [10], ZnO nanoparticles [11], copper [12], SiO_2_ nanoparticles [13,14], nanoclay [15], TiO_2_ [16], and Al_2_O_3_ and Fe_3_O_4_ nanoparticles [17,18]. Each of these additives has unique attributes, which significantly enhance the mechanical, thermal, or barrier properties of the composites.

A notable rise in the usage of graphene in polymer matrices research [19] can be attributed to graphene’s outstanding electrical, thermal, and mechanical properties. This novel material, which is formed from a single layer of sp2-bonded carbon atoms arranged in a repeating hexagonal pattern [20,21,22,23], has revolutionized various fields. Its applications include gas sensors [24], superconductors [25], catalysts [26], drug delivery systems [27], and material reinforcement [28], showcasing its versatility. Graphene’s superior properties are leveraged to enhance the performance of materials in these diverse applications. Despite these advancements, the synthesis of polymer nanocomposites is not without its challenges. The selection of an appropriate polymer matrix is critical. Polyolefins [29,30], despite being widely used, suffer from non-degradability. Polylactic acid [31,32], which is a biodegradable alternative, cannot be processed using water as a solvent [33,34,35]. In this scenario, polyvinyl alcohol (PVA) stands out as a polymer with a unique blend of characteristics. It occupies a middle ground between plastic and rubber, exhibiting excellent film-forming and adhesion properties, high chemical polarity, solubility in water, biodegradability, and a non-toxic nature. Furthermore, PVA is characterized by its outstanding chemical resistance and mechanical strength [36]. The integration of graphene into PVA nanocomposites, however, is limited and remains challenging.

The absence of functional groups in graphene complicates its dispersion within PVA, frequently resulting in subpar composite performance. For instance, Afzal and colleagues mixed graphene with PVA to create nanocomposite films and observed that the inclusion of graphene, regardless of whether microwave processing was employed, invariably led to diminished mechanical properties [37]. In addition, several studies focused on fabricating PVA composites with graphene, predominantly for the purpose of measuring conductivity [38,39,40], with minimal emphasis on the evaluation of mechanical performance due to the propensity of graphene to reduce the strength of PVA. Consequently, previous research largely concentrated on the use of graphene oxide (GO) for PVA nanocomposites because of its abundance of functional groups [41,42,43,44,45,46,47,48]. However, the high cost of GO presents a significant barrier to its scalability for industrial applications. Furthermore, its performance as a conductor is notably poor. Consequently, GO requires reduction to reduced graphene oxide (rGO) to enhance its conductivity, which introduces additional processing steps and costs. Moreover, the reduction process can lead to the loss of functional groups, thereby compromising its compatibility with PVA. Furthermore, research involving graphene in PVA primarily aimed to enhance conductivity [49], with mechanical properties and barrier performance being secondary considerations.

To address these limitations, this study introduces carboxy-functionalized graphene (CFG) as a cost-effective and functional alternative to standard graphene. CFG, which is enriched with carboxyl (COOH) groups and a reduced layer count of 10–30 layers, is anticipated to overcome the dispersion and cost challenges associated with conventional graphene. The study explores blending CFG with PVA to fabricate novel nanocomposite films. Ultrasonic vibration treatment is employed to ensure the homogeneous dispersion of CFG within the PVA matrix, aiming to achieve excellent compatibility between the two materials. The investigation focuses on evaluating the impact of CFG on various attributes of the PVA nanocomposites, including tensile properties, morphological characteristics, and barrier performance against oxygen and water vapor. The overarching goal is to unravel the potential of CFG/PVA nanocomposites for industrial applications, particularly in areas necessitating enhanced mechanical properties and superior barrier capabilities. This comprehensive study delves into the intricate interplay between CFG and PVA, aiming to elucidate the mechanisms by which CFG enhances the overall properties of the nanocomposites. The research is geared toward providing a blueprint for the development of advanced materials that can effectively meet the demands of modern applications, ranging from packaging and electronics to other sectors where enhanced material properties are paramount. By offering a detailed investigation into the properties and performance of CFG/PVA nanocomposites, this study will pave the way for future innovations in the field of polymer science and engineering.

## 2. Experimental Methods

### 2.1. Materials

For this research, all chemical reagents were of analytical grade and utilized without further purification. The polyvinyl alcohol (PVA), specifically type 1788, was procured from Shanghai Titan Scientific Co. Ltd., Shanghai, China. The carboxy-functionalized graphene (CFG) and graphene without a functional group were sourced from Lin-go Industrial Co., Ltd. (Zhongli, Taiwan), who are known for their specialization in the production of a custom grade of graphene.

### 2.2. Preparation of Nanocomposite CFG/PVA Films

**Figure 1:**
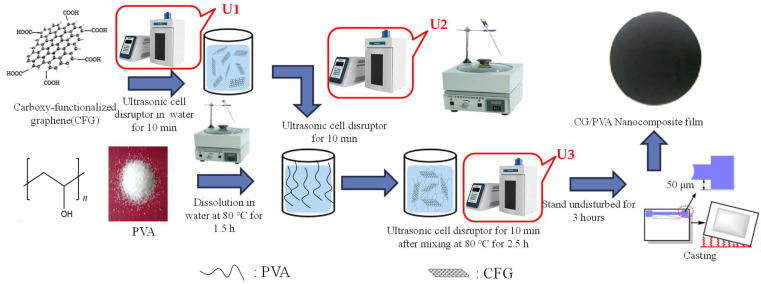
The fabrication of CFG/PVA nanocomposite films.

Initially, 17 g of PVA (Type 1788) was dissolved in 100 mL of water through heating and stirring on a magnetic stirrer for 1.5 h. CFG was subsequently added to a predetermined volume of water, with dispersion facilitated by an ultrasonic cell disruptor to distribute the nanofillers evenly. To determine the most suitable processing method, various ultrasonic treatments were applied to prepare the nanocomposite films, as illustrated in Figure 1. The treatments, designated U1, U2, and U3, were executed at different stages across four distinct processes (Processes 1 to 4), as shown in Table S1. Process 4 was ultimately selected as the processing condition for subsequent steps. A precise amount of CFG and graphene was then incorporated into the solution, achieving concentrations of 1%, 3%, and 6% relative to the PVA polymer concentration. This was followed by a second round of ultrasonic disruption to ensure a homogeneous mixture. The solution was further magnetically stirred for 2.5 h, and then subjected to a third dispersion via an ultrasonic cell disruptor. After allowing the mixture to settle for several hours, it was manually cast into films and dried for 3 h at a specified temperature, resulting in the fabrication of both CFG/PVA and graphene/PVA films. The compositions of all components are detailed in Table 1. The comprehensive procedure for the preparation of the nanocomposite film is depicted in Figure 1.

### 2.3. Mechanical Properties

To evaluate the tensile properties of both pure PVA and CFG/PVA nanocomposite films, we employed an electronic universal testing machine (Model FBS-10KNW, Xiamen Forbes Testing Equipment Co., Ltd.; Xiamen, China). The films were prepared with standardized dimensions of 50 mm in length and 2 mm in width. Prior to testing, each specimen was conditioned at 23 °C and 50% relative humidity for 24 h to ensure uniform environmental conditions. The testing procedure was conducted at a consistent crosshead speed of 10 mm/min. Mechanical property data were collected from at least five samples, and the mean of these results was computed. During the experiment, each film was securely clamped between the grips of the testing machine, ensuring uniform and aligned positioning to avoid any pre-stress or misalignment that could affect the test results. The force was applied until the film sample failed, and the tensile strength, elongation at break, and Young’s modulus were recorded for each sample. For the CFG/PVA nanocomposite films, a range of CFG concentrations was tested to investigate the impact of CFG content on the mechanical properties.

### 2.4. X-ray Diffraction (XRD) Analysis

For the structural characterization of graphene, CFG, pure PVA, and CFG/PVA nanocomposite films, X-ray diffraction analysis was carried out using a D2 Phaser X-ray diffractometer (Bruker Company, Ettlingen, Germany). This instrumental setup was chosen for its precision and reliability in detecting crystalline structures. The XRD spectra were obtained under the following conditions: an operating voltage of 40 kV and a current of 30 mA. The diffraction patterns were recorded over a range of 2θ angles from 5° to 60°, with a fine step size of 0.02° per second, ensuring detailed and accurate peak resolution.

### 2.5. Fourier Transform Infrared Spectroscopy (FTIR) Analysis

To discern the distinctions between carboxy-functionalized graphene (CFG) and graphene, and to highlight the presence of numerous functional groups in CFG, FTIR analysis was performed, covering the range from 500 to 3250 cm^−1^. Spectroscopy was employed to elucidate the chemical structure of the nanocomposites, with a particular focus on identifying the presence of C=O bonds, which are indicative of the reaction between CFG and PVA. The FTIR analysis was conducted within the wavenumber range of 2500 to 1500 cm^−1^, a spectrum known to be rich in information for identifying various functional groups, especially carbonyl groups. Prior to the FTIR test, sample preparation was meticulously carried out to ensure accurate results. The PVA and CFG/PVA film samples were carefully cut into small pieces that were suitable for FTIR analysis. These pieces were then thoroughly cleaned to remove any surface contaminants that could interfere with the infrared spectroscopy readings.

### 2.6. XPS Analysis

X-ray photoelectron spectroscopy (XPS) analysis was conducted using a Thermo Scientific K-Alpha+ spectrometer to investigate the surface composition, functional groups, and carbon atom hybridization levels of the sample. The sample, in powder form, was analyzed under a vacuum condition of less than 10–8 mbar using an Al Kα X-ray source at 100 W. High-resolution scans were performed at 50 eV. Binding energies were standardized based on the C1s and O1s peaks.

### 2.7. TGA Analysis

Thermogravimetric analysis (TGA) was conducted using a thermal analyzer (STA 409PC, Netzsch Company, Selb, Germany) under a nitrogen atmosphere. Differential thermogravimetry (DTG) analysis facilitated the measurement of changes in sample weight (weight loss rate) as the temperature was progressively increased. The experimental conditions included a temperature range of 25 to 550 °C and a heating rate of 10 °C per minute.

### 2.8. Evaluation of Water Vapor Barrier Properties

The water vapor transmission rate (WVTR) for the PVA and CFG/PVA nanocomposite films was assessed using a specialized testing apparatus, the W3/060 water vapor transmission test system, provided by Jinan Languang Electromechanical Technology Co., Ltd., Jinan, China. This equipment was selected for its precision and reliability in measuring water vapor transmission through various film materials. For the tests, the environmental conditions were meticulously controlled. The temperature was consistently maintained at 25 °C, and the relative humidity was set at 70%. Each film sample, with an approximate thickness of 1 mm, was securely mounted in the test chamber. The system was calibrated to ensure accurate and consistent readings. The testing protocol involved measuring the amount of water vapor passing through the film over a period of 30 min. WVTR data were collected from at least four samples, and the mean of these results was computed.

### 2.9. Oxygen Barrier Properties

To evaluate the oxygen transmission rates of both PVA and CFG/PVA films, a differential pressure gas permeameter (Model VAC-V2, Languang Electromechanical Technology Co., Ltd., Jinan, China) was utilized. This instrument measured the oxygen permeability in units of cm^3^/m^2^/day/Pa. The key testing conditions set for this analysis included maintaining the chamber temperature at 25 °C and using films with a thickness of 0.1 mm. The test environment comprised a mixture of oxygen and nitrogen gases, with an assessment ratio set at 10%. The relative humidity during testing was maintained at 55%. To ensure accurate measurements, the lower chamber of the device was degassed for 60 s, while both the upper and lower chambers underwent a degassing process for 2 h. The air pressure applied in the upper chamber was maintained at 1.01 kgf/cm^2^. Oxygen permeability measurements were taken from a minimum of three samples to calculate their average result.

### 2.10. Morphology Examination via Scanning Electron Microscopy

The morphologies of the PVA and CFG/PVA nanocomposite films were thoroughly examined using a Scanning Electron Microscope (SEM, model VEGA 3SBU, manufactured by TESCAN Company, Brno, Czech Republic). For optimal imaging, the samples underwent a gold sputtering process for a duration of 30 s, ensuring adequate conductivity for the SEM analysis. The operational parameters set for the SEM included an electric current of 5 mA and a scanning voltage of 3 kV, which are optimal for detailed surface imaging. Each sample prepared for the SEM observation measured 1.5 cm by 1.5 cm, which is a suitable size for ensuring comprehensive coverage of the material’s surface features during the scanning process.

### 2.11. Morphology Examinatio via Transmission Electron Microscopy

The morphology of the PVA and CFG/PVA nanocomposite films was analyzed using a Tecnai G2 F20 Transmission Electron Microscope (TEM) from FEI Company, Boston, MA, USA. Samples were prepared on a copper grid and observed under an accelerating voltage of 200 kV, achieving a point resolution of 0.24 nm and an information resolution of 0.14 nm, with the electron gun set to an energy resolution of ≤0.7 eV. This high-resolution imaging provided critical insights into the microstructural details of the nanocomposites, which are crucial for understanding the distribution and interaction of CFG within the PVA matrix.

### 2.12. Hydrophilicity

The hydrophilic nature of the nanocomposite films was evaluated using water contact angle tests, utilizing the JC2000D apparatus provided by Powereach in Shanghai, China. This analysis involved measuring the angle formed by a droplet of water on the film surface, which is indicative of the material’s affinity for water. For these measurements, the films were prepared with a standardized thickness of 1 mm, and the contact angle was recorded at intervals of 3 s, ensuring consistent and accurate assessment of the films’ surface wettability. Water contact angles were obtained from a minimum of five samples to calculate their average result.

### 2.13. Electrical Conductivity

The electrical conductivity properties of PVA and CFG/PVA nanocomposite films were investigated using a four-point probe resistivity testing technique. For this purpose, the RTS-8 Four-Probe Circular Discs instrument, manufactured by Four Probes Technology Co. Ltd. in Guangzhou, China, was employed. The film samples prepared for this assessment were circular in shape, each with a diameter of 4 cm and a thickness between 0.8 to 1.0 mm. To guarantee the precision of the results, a total of five conductivity measurements were taken at different locations on each film, with their average being used as the final conductivity value. The instrument’s current was regulated to a maximum of 10 µA for these tests. In addition, each film underwent a controlled heating process, using a sleeve to reach specific temperatures of 30 °C, 60 °C, and 120 °C before the conductivity was measured, thereby allowing an evaluation of the temperature-dependence of their electrical properties.

### 2.14. Antibacterial Test

The antibacterial properties of both PVA and CFG/PVA nanocomposite films were quantitatively assessed for their effectiveness against *E. coli*, following the methodology described by Tsou et al. [3]. In this testing protocol, samples of each film were submerged in serum bottles filled with an *E. coli* bacterial suspension. The bottles were then placed in an incubator, where they were maintained at a temperature of 37 °C and subjected to continuous agitation at a speed of 100 rpm over a 24-h period. The antibacterial performance of the films was evaluated by counting the number of *E. coli* colonies that formed after the incubation period, providing a clear measure of the capability of each film to inhibit bacterial growth. Data on antibacterial properties were derived from a minimum of three samples, and their average values were subsequently calculated.

## 3. Results and Discussion

### 3.1. Mechanical Properties

To identify the optimal processing parameters, ultrasonic treatments were applied at different stages, as depicted in Figure 1, and the tensile properties of the resulting films are documented in Table S1. The improved mechanical properties of CFG/PVA nanocomposites make them ideal candidates for applications that require robust materials, such as in packaging, and as part of structural components where durability and strength are crucial. The results reveal minimal differences between the U1 + U2 and U2 + U3 treatments, whereas the U1 + U3 process significantly underperforms in tensile properties. This outcome highlights the critical importance of the U2 treatment. The introduction of ultrasonic treatment at various stages can affect the dispersion effectiveness, thereby influencing the mechanical properties of the produced nanocomposite films. However, as shown in Table S1, films prepared using Process 4 (U1 + U2 + U3) exhibit superior performance. Consequently, nanocomposite films incorporating various concentrations of CFG and graphene were prepared following Process 4.

**Figure 2:**
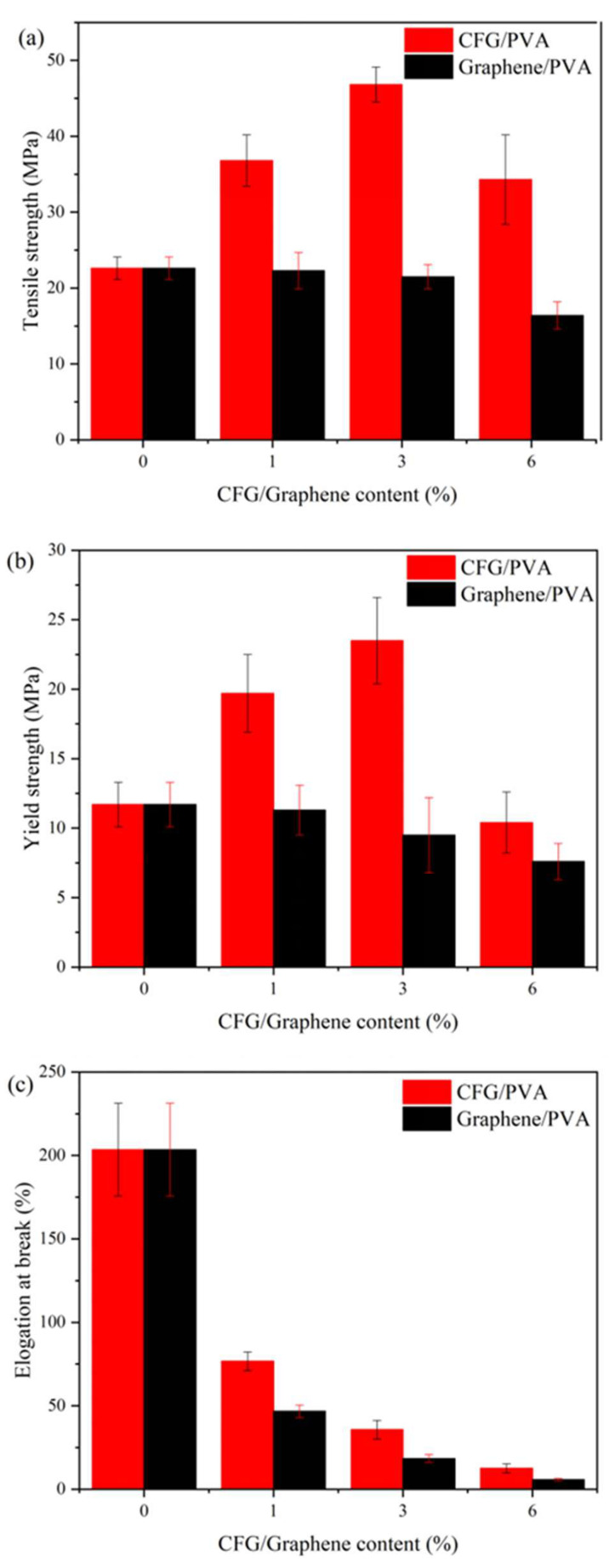
PVA and nanocomposite CFG/PVA films: (**a**) tensile strength; (**b**) elongation at break; (**c**) yield strength.

Figure 2 demonstrates how different concentrations of CFG and unfunctionalized graphene impact the tensile strength and elongation at break of CFG/PVA and graphene/PVA nanocomposite films. The addition of graphene not only fails to enhance the performance of PVA but also results in a reduction of the nanocomposite films’ tensile properties, consistent with the findings of Afzal et al. [37]. This could be due to the absence of functional groups on graphene preventing interaction with PVA. In addition, graphene’s hydrophobic nature contrasted with PVA’s hydrophilicity, leading to graphene aggregation within the PVA matrix. Data analysis shows a distinct improvement in both tensile and yield strengths with CFG addition, despite a decrease in elongation at break. Incorporating just 1% CFG into PVA significantly enhances mechanical strength. Moreover, a nanocomposite with 3% CFG content displays optimal mechanical properties [50,51,52,53], as detailed in Figure 2. The tensile strength and yield strength of the 3% CFG/PVA nanocomposite are approximately 2.07 and 2.01 times higher, respectively, than those of pure PVA. The data in Table S1 indicate that increasing the CFG content leads to a marked improvement in tensile and yield strengths, especially at the 3% CFG level. However, at higher concentrations, particularly at 6% CFG, there is a noticeable decline in mechanical properties. This reduction is attributed to the aggregation of CFG particles at higher concentrations, which disrupts the uniformity and continuity of the polymer matrix. As a result, although the nanocomposite gains in strength, its flexibility and elongation at break are compromised by the agglomeration effect.

These findings emphasize the necessity of balancing CFG concentration to optimize mechanical properties without sacrificing the material’s ductility. Excessive CFG causes particle agglomeration, which adversely affects the overall mechanical performance of the nanocomposite film. Therefore, critically evaluating the optimal CFG concentration is crucial for achieving the desired balance between strength and flexibility in CFG/PVA nanocomposites for practical applications.

### 3.2. X-ray Diffraction (XRD) Analysis

**Figure 3:**
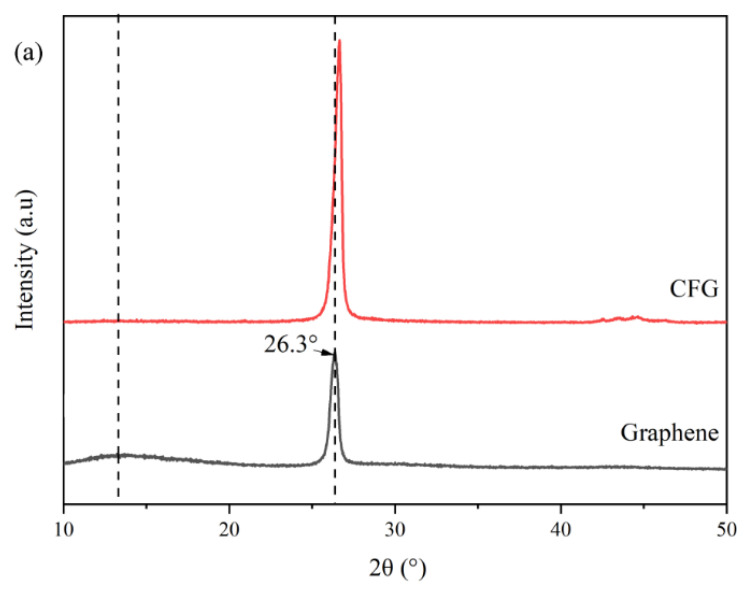

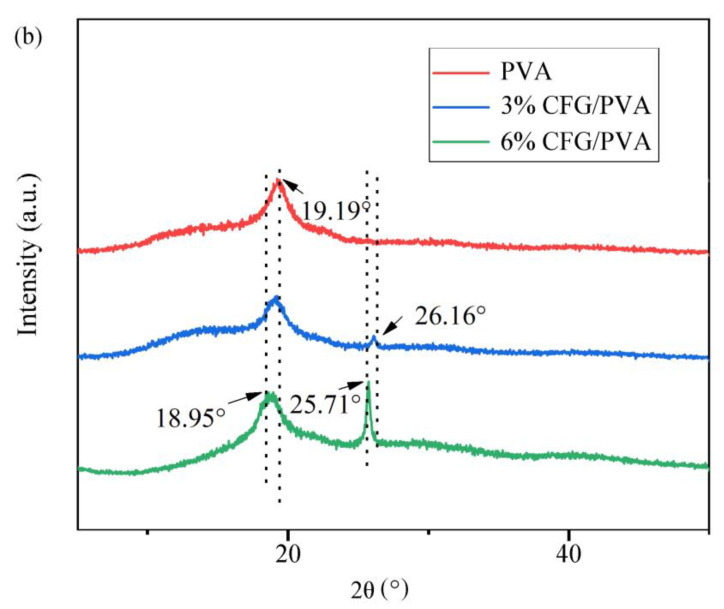
XRD patterns: (**a**) CFG and graphene; (**b**) PVA and CFG/PVA films.

To further understand the distinctions between CFG and graphene, we conducted XRD analysis on both nanomaterials, as depicted in Figure 3a. The analysis revealed that the primary characteristic peak for both materials occurred at 2θ = 26.3°; however, CFG exhibited a notably higher peak intensity compared with graphene. This enhancement in peak intensity for CFG could be attributed to the introduction of carboxyl functional groups, which may facilitate a more ordered stacking between graphene layers. These functional groups are capable of forming regular spacings through non-covalent interactions such as hydrogen bonding, potentially improving the material’s crystallinity. Considering the tensile performance results, we selected CFG with its higher crystallinity as the primary focus for this study, adding it to PVA for further investigation.

The results of XRD analysis of pure PVA and CFG/PVA nanocomposite films are depicted in Figure 3b. For pure PVA, distinct diffraction peaks are observed at 2θ angles of 19.18° and 18.95°, which are characteristic of the crystalline structure of PVA [54]. Upon the addition of 3% CFG to PVA, a new diffraction peak emerges at 2θ = 26.17° [55], aligning closely with the primary diffraction peak of CFG, indicating the presence and influence of CFG in the composite structure. Interestingly, increasing the CFG content to 6% results in a noticeable shift of this peak from 26.17° to 25.71°. According to Bragg’s law, a larger 2θ value correlates to a smaller interplanar spacing [56]. This shift suggests that the aggregation of CFG at higher concentrations leads to subtle changes in the crystal structure of the CFG/PVA composite. Furthermore, the intensified diffraction peak at higher CFG concentrations could be attributed to the partial exposure of CFG on the surface of PVA due to severe agglomeration. This exposure results in a more pronounced diffraction peak compared with the intrinsic peaks of PVA.

These XRD findings indicate that although the incorporation of CFG into PVA can alter and potentially enhance its crystalline structure, there is a critical limit for the CFG content. Beyond this limit, excessive nanofiller aggregation not only alters the crystalline structure but may also adversely affect the composite material’s overall homogeneity and performance. Therefore, optimizing the concentration of CFG is crucial to maintain a balance between enhancing the material properties and preserving the structural integrity of the CFG/PVA nanocomposite.

### 3.3. FTIR

**Figure 4:**
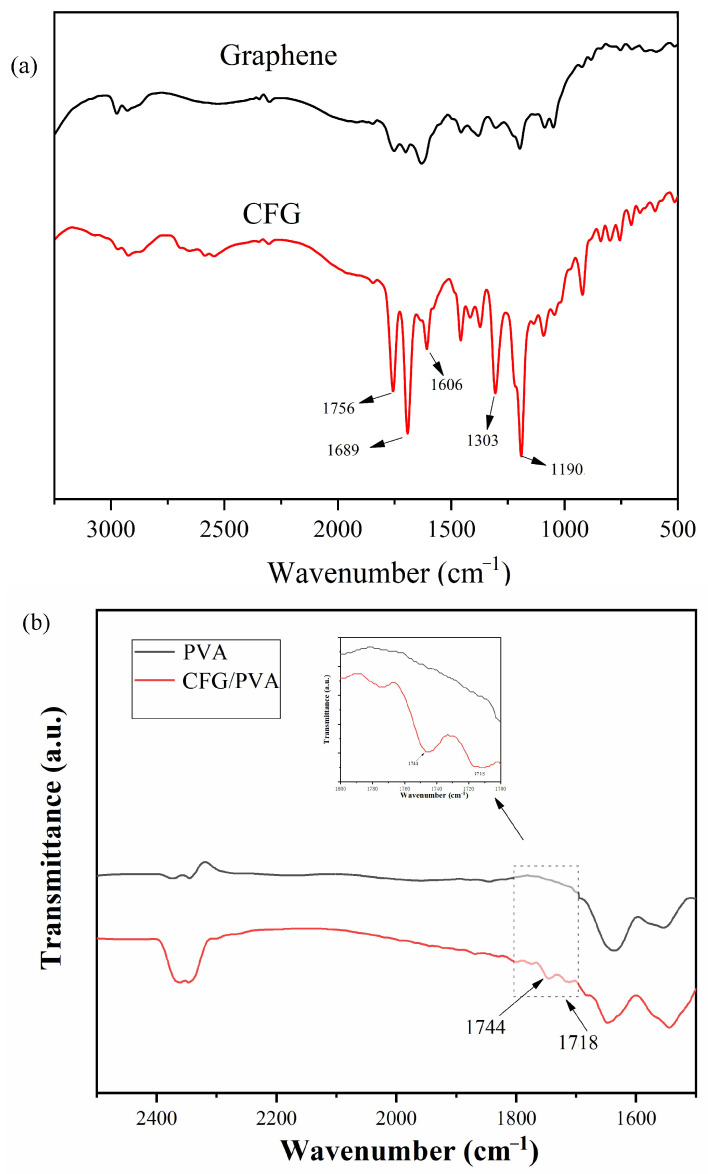
FTIR spectra of (**a**) Graphene and CFG; (**b**) PVA and nanocomposite CFG/PVA film.

We conducted FTIR analysis to compare the differences between CFG and graphene, and to demonstrate the abundant functional groups present in CFG. Figure 4a presents the results of FTIR analysis of CFG and graphene. The figure shows that graphene exhibits no significant peaks owing to the lack of functional groups in its pure form. Conversely, CFG displays distinct and sharp characteristic peaks at 1756, 1689, 1606, 1303, and 1190 cm^−1^. The peak at 1756 cm^−1^ is typically attributed to the C=O stretching vibration of carboxylic acid groups (-COOH), making it a typical characteristic peak of carboxyl groups for CFG [57,58]. The peak at 1689 cm^−1^ may indicate C=O stretching vibrations, located slightly below 1756 cm^−1^, potentially due to interactions between functional groups or conjugation effects. This could also suggest the presence of partially oxidized carboxylic esters (-COOR) or carbonyl groups (C=O). In addition, the peaks at 1303 cm^−1^ and 1190 cm^−1^ are attributed to C-O vibrations in carboxylic acid groups, with the peak at 1190 cm^−1^ also being related to C-O stretching vibrations, likely from C-O-C vibrations in carboxyl or carboxylic ester groups [59]. These results demonstrate that, compared with graphene, CFG is rich in carboxyl groups, thereby enhancing compatibility with PVA. Therefore, this study opts to incorporate CFG into PVA to fabricate nanocomposite films.

Figure 4b reveals key insights into the chemical interactions within neat PVA and CFG/PVA nanocomposite films. The notable absorption peak at approximately 1718 cm^−1^ and 1744 cm^−1^ in the CFG/PVA spectrum is indicative of the C=O vibration in the ester bond. This bond is formed by the reaction between the carboxylic group in CFG and the hydroxyl group in PVA. This observation aligns with data reported in previous studies [60,61], confirming the successful formation of ester bonds in the composite material. This structural arrangement is crucial for enhancing the overall performance of the material. The results of FTIR analysis confirm that the addition of CFG significantly enhances the mechanical strength.

### 3.4. X-ray Photoelectron Spectroscopy (XPS)

**Figure 5:**
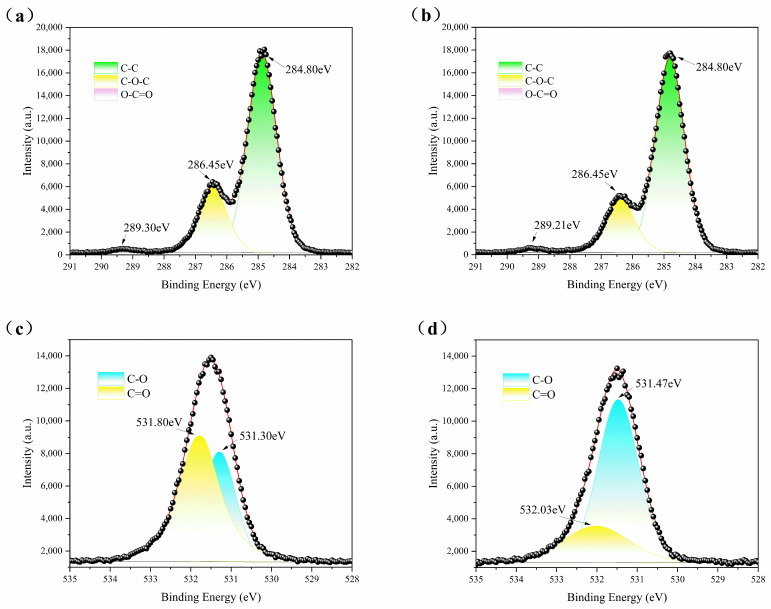
XPS analysis: (**a**) C1s for PVA; (**b**) C1s for CFG/PVA; (**c**) O1s for PVA; (**d**) O1s for CFG/PVA.

XPS analysis was employed to further substantiate the chemical reaction between CFG and PVA, and the results are depicted in Figure 5. Figure 5a,b respectively illustrate the C1s spectra of PVA and CFG/PVA nanocomposite films. Within these spectra, the positions of the peaks at 284.8 eV representing C-C bonds and at 286.45 eV for C-O-C bonds remained unchanged, but the intensity of the C-O-C bond peaks decreased by approximately 30%. This indicates the successful integration of CFG into the PVA matrix without inducing significant chemical alterations in the C-O-C bond structure. Conversely, the peak specific to PVA at 289.3 eV for O-C=O shifted to 289.21 eV upon the addition of CFG, likely owing to an esterification reaction between the carboxyl functional groups of CFG and the hydroxyl groups in PVA, resulting in the formation of new ester bonds (O-C=O). This slight peak shift reflects changes in the chemical environment, consistent with a chemical interaction between CFG and PVA that leads to cross-linked structures within the nanocomposite films [62].

Further examination of the O1s spectra for PVA and CFG/PVA composite films, as shown in Figure 5c, and 5d, reveals a peak at 531.3 eV (C-O) for pure PVA film, which shifts to 531.47 eV in the CFG/PVA nanocomposite film, accompanied by a significant increase of approximately 40% in peak intensity. This notable increase in intensity and shift suggests that the inclusion of CFG altered the oxygen-containing chemical environment in PVA, particularly the hydroxyl environment associated with C-O. Moreover, the peak in pure PVA film at 531.8 eV for C=O, upon the addition of CFG, shifted to 532.03 eV, with a marked reduction in peak intensity by about 120%. This shift may be attributed to the esterification reaction between the carboxyl groups of CFG and the hydroxyl groups of PVA, not only altering the chemical environment but potentially leading to a redistribution of electron density around the C=O bond. The esterification reaction, which results in a decrease in hydroxyl groups, thereby increases the C=O bond content, and this change is manifested in the O1s spectra through peak shift and intensity increase. However, the significant reduction in peak intensity could be due to the inclusion of CFG altering the chemical environment of C=O functional groups in PVA, thereby affecting their electron cloud density. The carboxyl groups of CFG reacting with PVA’s hydroxyl groups reduce the number of free C=O functional groups detectable by XPS, significantly decreasing the corresponding intensity of the peak [63]. These XPS analysis results further support the hypothesis of a chemical interaction between CFG and PVA, specifically an esterification reaction, leading to the formation of cross-linked structures within the nanocomposite films. This chemical cross-linking not only enhances the mechanical properties of the nanocomposite films but may also positively impact other properties, such as barrier performance and thermal stability.

**Figure 6:**
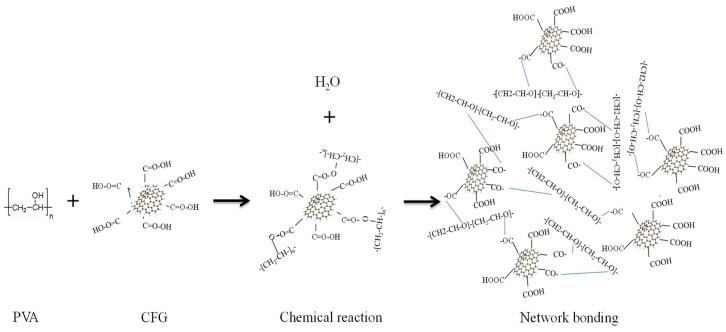
Chemical reaction mechanism for nanocomposite CFG/PVA film.

The reaction mechanism between PVA and CFG, depicted in Figure 6, illustrates the development of a three-dimensional network structure within the composite. The formation of ester bonds between CFG and PVA leads to a more interconnected and robust molecular structure, which imparts improved mechanical properties. This enhanced structural integrity is likely responsible for the increased tensile strength and yield strength observed in the CFG/PVA nanocomposites. Furthermore, the three-dimensional network structure contributes to a reduction in the permeability of water vapor and oxygen, enhancing the barrier properties material. These improvements are critical for applications in packaging where moisture and oxygen resistance are essential. In addition, the interaction between CFG and PVA reduces the number of hydrophilic groups, increasing the material’s hydrophobicity. This change in the surface chemistry of the composite film may make it less prone to water absorption, further enhancing its suitability for use in environments where moisture resistance is paramount.

### 3.5. TGA

**Figure 7:**
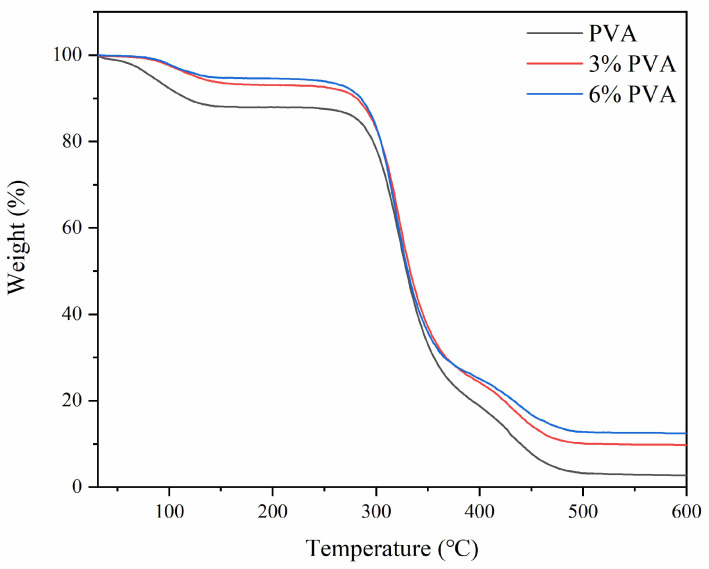
The TGA curve of PVA and CFG/PVA nanocomposite.

The potential for covalent bonding between PVA and CFG can significantly enhance the thermal stability of the nanocomposite material. Therefore, we conducted TGA tests on both PVA and CFG/PVA nanocomposite materials. The TGA curves are presented in Figure 7. Compared with the thermal stability of pure PVA, the onset temperatures of weight loss for CFG/PVA nanocomposite films increased with the addition of CFG. This trend was consistent across the temperatures at 5% (T5) and 10% (T10) weight loss. This improvement is likely due to the ester bonds formed between the hydroxyl groups in PVA and the carboxyl groups in CFG [64,65], which enhance the material’s resistance to thermal decomposition by increasing intermolecular interactions. Moreover, the formation of ester bonds can also impede the internal transfer of thermal energy, as this dense cross-linked network restricts the movement of molecular chains, further enhancing thermal stability. In addition, the layered structure of CFG may act as a thermal barrier [66], further inhibiting the propagation of heat and protecting the PVA matrix from premature thermal decomposition.

These findings corroborate the insights provided by FTIR and XPS analyses, which indicate that the COOH groups in CFG can form ester bonds with the OH groups in PVA. The formation of these covalent bonds not only enhances the thermal stability of nanocomposite film but may also positively impact its mechanical properties. The introduction of ester bonds can increase the rigidity and strength of the nanocomposite material, thereby enhancing its load-bearing capacity and durability. Furthermore, the carboxyl groups in CFG can improve the dispersibility and interfacial compatibility of the PVA matrix, further boosting the overall performance of the nanocomposite material.

### 3.6. Water Vapor Barrier Properties

**Figure 8:**
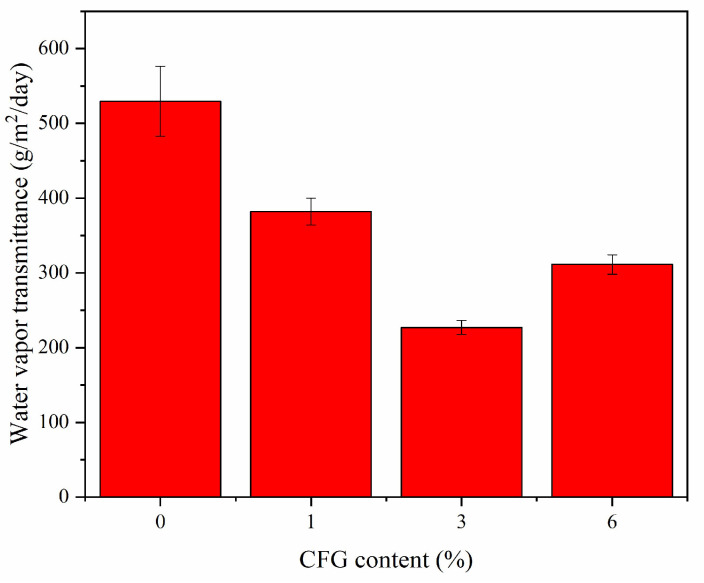
Water vapor barrier properties of PVA and CFG/PVA films.

Figure 8 illustrates the water vapor barrier properties of both pure PVA and CFG/PVA nanocomposite films. Notably, the introduction of just 1% CFG into PVA caused a decreasing trend in water vapor transmission. The reduction in permeability reached its maximum at a 3% CFG concentration, where the nanocomposite film demonstrated its best water vapor barrier performance. As depicted in Table 2, the water vapor transmittance for the CFG/PVA film was significantly lower compared with that of pure PVA, indicating an enhanced barrier effect.

However, an interesting observation was made when the CFG content was increased to 6%. At this higher concentration, the water vapor permeability for CFG/PVA increased, contrary to the expected trend. This increase in permeability can be attributed to the excessive amount of CFG, which is prone to agglomeration within the film matrix [50,67,68,69]. The aggregation of CFG particles at higher concentrations disrupts the uniformity of the film, creating pathways that increase water vapor transmission. This phenomenon highlights the critical balance needed in nanofiller concentration to optimize the barrier properties of the film. Too little CFG may not impart sufficient barrier characteristics, whereas too much can lead to aggregation and compromise the film’s integrity and performance. Thus, determining the optimal CFG content is essential for achieving the desired balance between enhanced barrier properties and maintainable film uniformity and stability.

### 3.7. Oxygen Barrier Properties

**Figure 9:**
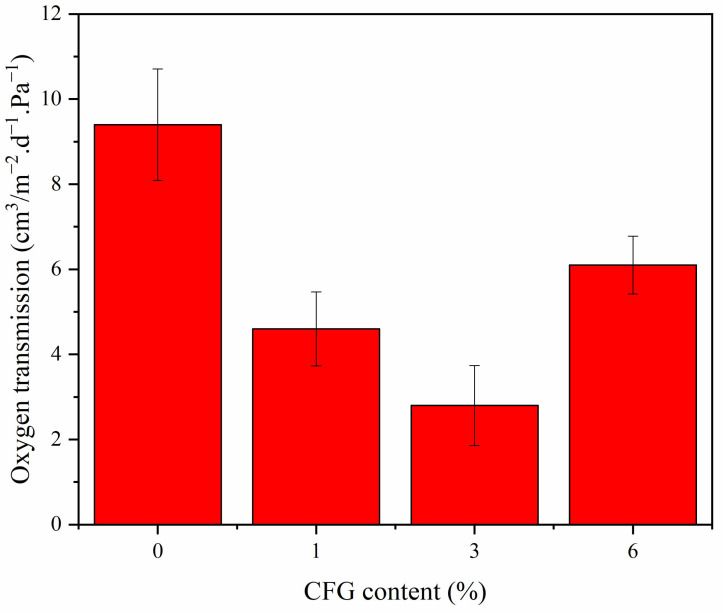
Oxygen barrier properties of PVA and nanocomposite CFG/PVA films.

**Figure 10:**
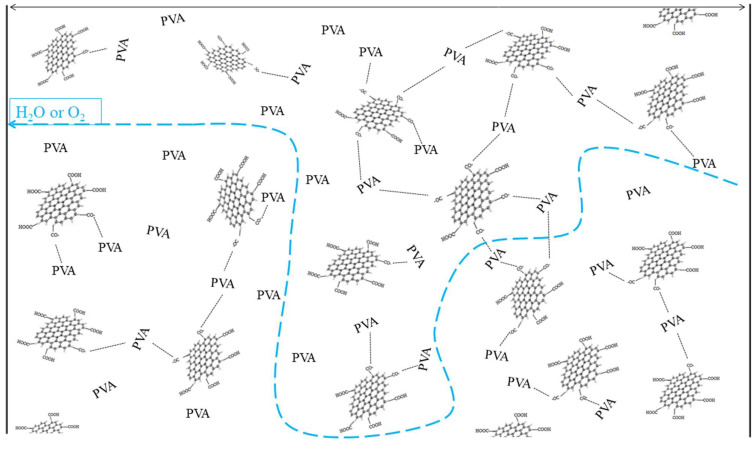
Schematic of the molecular permeation path for water vapor or oxygen.

Figure 9 presents the oxygen transmittance (OT) test results for both pure PVA and CFG/PVA nanocomposite films. Analysis of the oxygen permeability data for CFG/PVA films revealed a substantial enhancement in oxygen barrier properties, even with just a 1% addition of CFG, reducing the OT value by more than half. In particular, the optimal oxygen barrier performance was achieved with a 3% CFG content. As detailed in Table 2, the OT value dropped significantly from 9.4 cm^3^/m^2^·d·Pa to 3.4 cm^3^/m^2^·d·Pa. These results underscore the role of CFG in markedly boosting the oxygen resistance of PVA films [70]. This improvement is attributed to the nanomaterials creating more tortuous pathways for water vapor and oxygen to traverse through the film, as illustrated in Figure 10. However, when the CFG content was increased to 6%, there was a noticeable increase in the OT of the film. This can be ascribed to the overabundance of CFG, which tends to agglomerate and pose challenges in dispersion during film preparation and formation. Research on PVA-based graphene nanocomposite films for barrier properties is scarce. A comparison with other related studies is presented in Table 2, where the oxygen barrier performance of this work surpasses that reported in other publications [71,72,73,74,75]. None of these studies documented the water vapor barrier properties. Consequently, the excessive CFG content adversely impacts the oxygen resistance capabilities of the nanocomposite film. Therefore, it is crucial to optimize CFG concentration in the PVA matrix to balance the enhancement in barrier properties with the practicalities of film processing [76,77].

**Table 2:** Comparison of oxygen barrier performance of various nanocomposite PVA films.

Sample	Oxygen Barrier Performance (%)		Water Vapor Barrier Performance (%)		Reference
	Oxygen Transmission (cm^3^/m^−2^·d^−1^·Pa^−1^)	Permeability Reduction Factor (Times)	Water Vapor Transmission (g/m^2^/day)	Permeability Reduction Factor (Times)	
PVA/CFG	2.8	3.36	226.8	2.2	This work
PVA/GO	4	2.225	n/a	n/a	[71]
PVA/GO	107.14	1.07	n/a	n/a	[72]
PVA/GO/CuSO_4_ 5H_2_O	4.2	2.476	n/a	n/a	[73]
PVA/rGO	2.98	2.48	n/a	n/a	[74]
PVA/CNF/GO	3	1.65	n/a	n/a	[75]

### 3.8. Scanning Electron Microscope (SEM) Analysis

**Figure 11:**
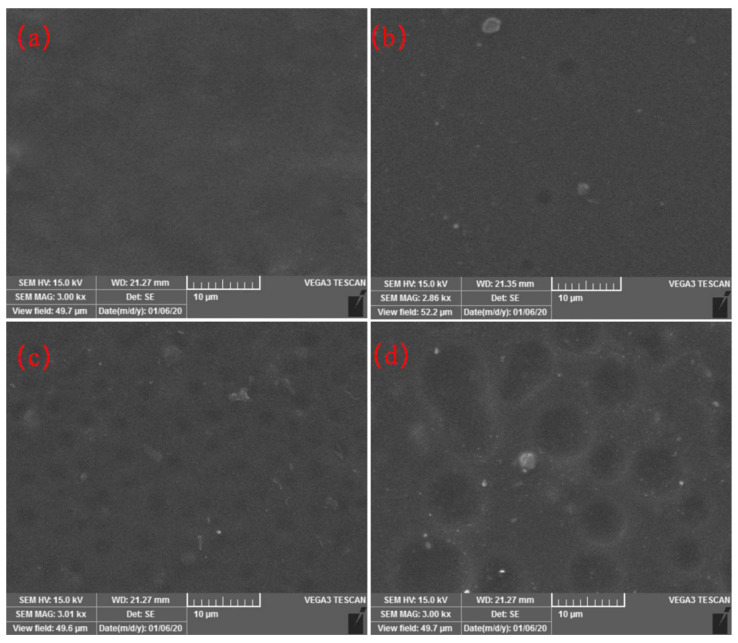
SEM images of PVA and nanocomposite CFG/PVA films: (**a**) PVA; (**b**) 1%CFG/PVA film; (**c**) 3%CFG/PVA film; (**d**) 6%CFG/PVA film.

SEM analysis of PVA and CFG/PVA nanocomposite films offers an insightful examination into their surface morphology, as depicted in Figure 11. The tensile fracture surface of the pure PVA film (Figure 11a) exhibits a remarkably smooth texture that is indicative of the homogenous nature of the polymer. The incorporation of 1% CFG into the PVA matrix (Figure 11b) introduces slight surface undulations and reveals particle-like or flake-like features, signifying a uniform dispersion of CFG. In addition, shallow circular indentations are observed, potentially resulting from the debonding of CFG after tensile testing, leaving behind defects. With an increase in CFG content to 3% (Figure 11c), the undulations become more pronounced, and the circular indentations are more abundant and uniformly distributed across the surface. The presence of these features, along with an increased number of particle-like and flake-like elements, suggests that the CFG is adequately and still evenly distributed throughout the PVA matrix. The spaces left by CFG debonding after tensile fracture significantly bolster the polymer matrix, leading to notable improvements in mechanical properties, such as enhanced tensile and yield strengths. However, at a CFG concentration of 6% (Figure 11d), the previously small circular indentations evolve into larger and more inconsistent circular grooves with significant depth variations. This irregularity likely stems from the excessive CFG content, which causes uneven distribution and noticeable agglomeration, thereby adversely affecting the mechanical strength.

These findings reveal that a low to moderate level of CFG can augment the mechanical properties of PVA films through uniform dispersion and effective reinforcement. Conversely, an excessive amount of CFG leads to detrimental effects owing to agglomeration and uneven distribution. The tensile performance data indicate that as the CFG content increases, the tensile strength and yield strength of the PVA nanocomposite films improve significantly, particularly exhibiting optimal mechanical properties at a CFG content of 3%. This observation aligns with the results of SEM analysis, suggesting that the uniform dispersion and effective reinforcement of CFG play a crucial role in enhancing the mechanical strength of the film. However, beyond a CFG content of 3%, the mechanical properties decline owing to excessive agglomeration and uneven distribution of CFG, which is also reflected in the tensile performance test data. Therefore, it is imperative to optimize the CFG concentration to maximize the mechanical performance of PVA/CFG nanocomposite films, ensuring a balance between strength enhancement and material homogeneity.

### 3.9. Transmission Electron Microscope (TEM) Analysis

**Figure 12:**
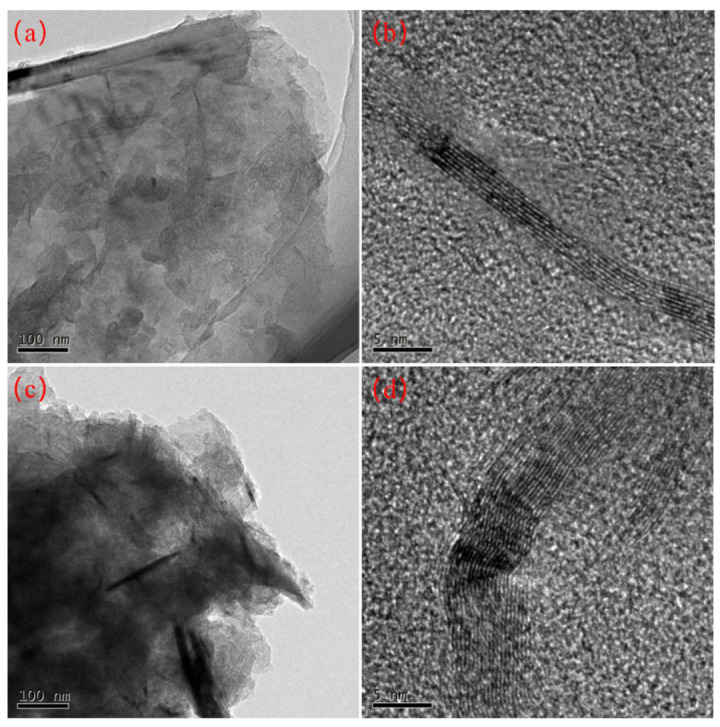
TEM images of nanocomposite CFG/PVA films: (**a**,**b**) 3%CFG/PVA film; (**c**,**d**) 6%CFG/PVA film.

Transmission Electron Microscope (TEM) images of the nanocomposite films, presented in Figure 12 at identical magnifications, reveal the effects of ultrasonic vibration treatment. The PVA film with a 3% CFG concentration shows effective dispersion, evidenced by the relatively transparent appearance in Figure 12a. A closer examination at a higher magnification (Figure 12b) reveals that there are fewer than 10 (approximately 8) CFG layers. Conversely, the PVA film with a 6% CFG concentration appears more opaque and darker, indicating noticeable aggregation due to its higher concentration, as observed in Figure 12c. This aggregation leads to a significant increase in the number of layers, with close to 20 layers visible upon closer inspection in Figure 12d.

The TEM results not only confirm that CFG can enhance the oxygen and water vapor barrier properties of PVA but also highlight a critical concentration threshold for effective dispersion. At 3% CFG, the nanofiller is well-distributed within the PVA matrix, contributing to improved barrier properties and maintaining the film’s mechanical integrity. In contrast, at a 6% CFG concentration, the tendency for aggregation becomes pronounced. This excessive aggregation leads to the formation of defects within the nanocomposite film, which adversely affects the tensile strength and overall barrier performance of the PVA [78,79]. These findings underscore the importance of optimizing CFG content in PVA to balance enhanced barrier properties with maintaining the structural integrity of the nanocomposite film.

### 3.10. Hydrophilicity

**Figure 13:**
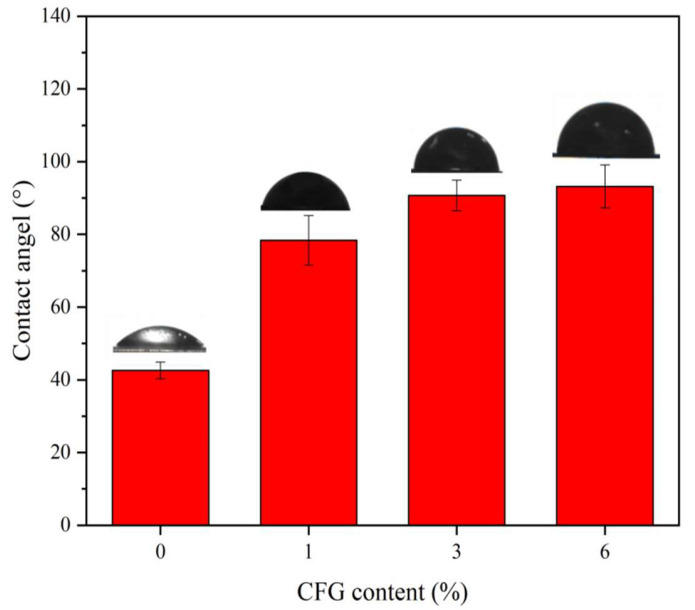
Contact angle of PVA and nanocomposite CFG/PVA films at 3 S.

To verify whether CFG enhances water resistance, contact angle analyses were conducted on all samples. Contact angles serve as indicators of the hydrophilic or hydrophobic characteristics of nanocomposite films. As illustrated in Figure 13, the contact angle of the PVA film increased significantly with the addition of CFG, demonstrating that CFG effectively enhances the hydrophobicity of PVA. Specifically, with a 1% addition of CFG, the contact angle increased from 42.6° to 78.4°. Furthermore, when the CFG content was increased to 3% and 6%, the contact angles exceeded 90°, which is generally regarded as the threshold between hydrophilicity and hydrophobicity in materials. This transition to hydrophobicity can be attributed to the formation of ester bonds between the COOH groups in CFG and the OH groups in PVA. This reaction reduces the number of hydrophilic groups and introduces more hydrophobic ester linkages [50,80]. The incorporation of CFG, therefore, not only diminishes the hydrophilicity of PVA but also contributes to its improved resistance to water vapor, elucidating the mechanism behind CFG’s enhancement of the barrier properties of PVA [81]. This finding is significant, as it provides a clear understanding of how CFG alters the surface characteristics of PVA, offering potential for tailored applications where controlled hydrophobicity is desired.

### 3.11. Conductivity

**Figure 14:**
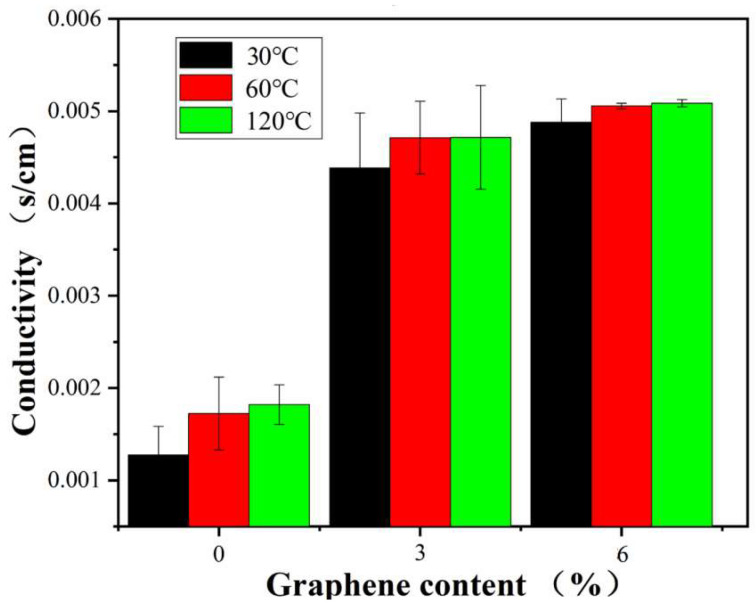
Conductivity of PVA and nanocomposite CFG/PVA films at different temperatures.

Given that graphene’s high electrical conductivity can endow polymers with electrical conductive properties, we conducted tests to understand the impact of CFG on the electrical conductivity of PVA. Figure 14 presents the electrical conductivity measurements of pure PVA and CFG/PVA nanocomposite films across a range of temperatures. The findings reveal a notable increase in the conductivity of the nanocomposites with the incorporation of CFG, highlighting the role of CFG in enhancing electrical properties. Interestingly, the conductivity levels of the nanocomposite films containing 3% and 6% CFG were similar. This phenomenon can be attributed to the saturation of conductive pathways at higher CFG concentrations, suggesting that beyond a certain CFG content, no additional structural continuity improvements are achieved between CFG nanoparticles.

Furthermore, a consistent upward trend in conductivity was observed as the temperature increased from 30 to 120 °C. This increase in conductivity at elevated temperatures can be explained by a reduction in contact resistance among graphene particles or enhanced connectivity within the graphene network [82,83,84,85,86]. Such temperature-dependent conductivity behavior indicates that CFG significantly contributes to the electrical performance of the nanocomposite films at both lower and higher temperature ranges.

These results not only demonstrate that CFG improves the electrical conductivity of PVA-based nanocomposites but also suggest that there is an optimal CFG content threshold for achieving maximum conductivity enhancement. Above this threshold, additional CFG does not further improve conductivity, likely owing to aggregation and saturation effects. The temperature-responsive conductivity of these nanocomposites also opens up potential applications in temperature-sensitive electronic devices where material conductivity needs to adapt to changing thermal environments. Thus, CFG/PVA nanocomposites with optimized CFG contents could offer valuable solutions in the field of flexible electronics and smart materials.

#### 3.12. Antibacterial Properties

**Figure 15:**
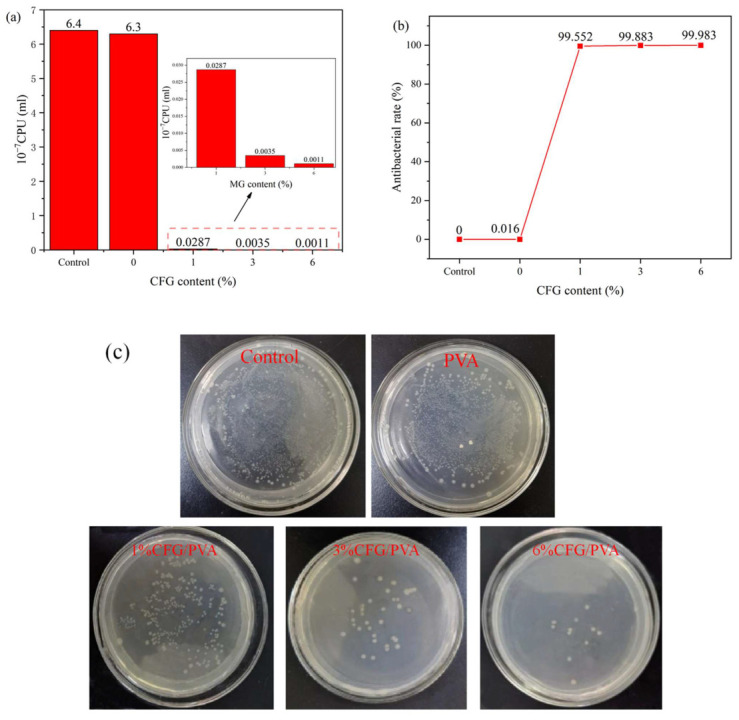
Antibacterial effectiveness of PVA and nanocomposite CFG/PVA films: (**a**) number of colonies; (**b**) antibacterial rate; (**c**) images showing the number of *E. coli* resistant colonies in nanocomposites.

Graphene has been reported to possess antibacterial properties. If these properties could be imparted to PVA, it would broaden its applications. Therefore, we conducted antibacterial assessments on the samples. Figure 15a provides a comprehensive evaluation of the antibacterial effectiveness of PVA and CFG/PVA nanocomposite films against *E. coli* over a 24-h period. The data vividly illustrate the significant impact of CFG on enhancing the antibacterial properties of the nanocomposite films. Figure 15b calculates the antibacterial rate using the control group. Figure 15c shows images of the number of *E. coli* resistant colonies in nanocomposites. Notably, with the addition of CFG, a drastic improvement in antibacterial activity is observed, with the films containing 3% or more CFG displaying an inhibition rate exceeding 99.86%. This remarkable increase in antibacterial efficiency is a direct consequence of the presence of CFG in the films [87,88]. The striking inhibition rates of 99.552%, 99.883%, and 99.983% for the 1%, 3%, and 6% CFG/PVA nanocomposites, respectively, underscore the potent antimicrobial action imparted by CFG. The control and pure PVA samples exhibit negligible antibacterial activity, with rates close to 0%. However, as CFG content increases, the antibacterial rate soars, significantly reducing the *E. coli* bacterial counts.

This pronounced antibacterial property of the CFG/PVA nanocomposites can be attributed to the unique structural and chemical characteristics of CFG, which disrupt bacterial cell walls and prevent their proliferation. The sharp edges of CFG can directly cut through bacterial cell walls, leading to the leakage of cellular contents and resulting in cell death. Furthermore, CFG can induce the production of reactive oxygen species, which can damage the bacterial cell membrane and subsequently harm the internal structure and function of the cell, leading to bacterial death [89,90].

The results indicate that even a small addition of CFG (1%) dramatically enhances the antibacterial capacity of the films. This enhancement becomes more pronounced with higher CFG concentrations, making these nanocomposites highly effective against *E. coli* contamination. The findings from this study suggest that CFG/PVA nanocomposites are highly promising materials for applications where antibacterial properties are crucial, such as in food packaging. By incorporating CFG, these films can significantly reduce the risk of foodborne illnesses caused by *E. coli*, ensuring safer food preservation and extending the shelf life of perishable products. Consequently, the use of CFG/PVA nanocomposites in food packaging may represent a significant advancement in maintaining food hygiene and safety standards.

## 4. Conclusions

This comprehensive study conclusively demonstrates that CFG significantly enhances the mechanical, barrier, electrical, and antibacterial properties of PVA nanocomposites, with the most notable improvements being observed at a 3% CFG concentration. Beyond this optimal concentration, the benefits are overshadowed by the adverse effects of CFG aggregation on the composite’s properties. Detailed chemical analysis using FTIR and XPS verified the formation of ester bonds between CFG and PVA, a key factor in the material’s enhanced performance. Morphological assessments via scanning and transmission electron microscopy revealed that a 3% concentration of CFG ensures uniform dispersion within the PVA matrix, which is crucial for achieving superior composite properties. The study highlights the pivotal role of CFG in improving the thermal stability and hydrophobicity of the nanocomposites, thereby enhancing their resistance to moisture and extending their application potential in fields that require durable and moisture-resistant materials. Furthermore, the increased electrical conductivity and exceptional antibacterial properties introduced by the addition of CFG expand the utility of PVA nanocomposites into electronics and packaging, where such functionalities are increasingly demanded. Overall, this research underscores the importance of optimizing CFG concentration within PVA nanocomposites to fully harness their potential across a broad spectrum of industrial applications, thereby offering a significant step forward in the development of advanced materials technology.

## Figures and Tables

**Table 1:** The components of PVA, CFG/PVA, and graphene/PVA nanocomposites.

Sample	PVA (%)	CFG (%)	Graphene (%)
PVA	100	0	0
1% CFG/PVA	99	1	0
3% CFG/PVA	97	3	0
6% CFG/PVA	94	6	0
1% graphene/PVA	99	0	1
3% graphene/PVA	97	0	3
6% graphene/PVA	94	0	6

## Data Availability

The data are not publicly available due to contain proprietary data that are part of ongoing studies.

## Supplementary Materials

The following supporting information can be downloaded at: https://www.mdpi.com/article/10.3390/polym16081070/s1, Table S1: Mechanical properties of PVA and nanocomposite 3%MG/PVA.

## References

[1] Tsou C.H., Wu C.S., Hung W.S., De Guzman M.R., Gao C., Wang R.Y., Suen M.C. (2019). Rendering polypropylene biocomposites antibacterial through modification with oyster shell powder. Polymer.

[2] Luo X.L., Xu J.J., Wang J.L., Chen H.Y. (2005). Electrochemically deposited nanocomposite of chitosan and carbon nanotubes for biosensor application. Chem. Commun..

[3] Tsou C.H., Yao W.H., Lu Y.C., Tsou C.Y., Wu C.S., Chen J., Wang R., Su C.C., Hung W.S., De Guzman M.R. (2019). Antibacterial property and cytotoxicity of a poly (lactic acid)/nanosilver-doped multiwall carbon nanotube nanocomposite. Polymers.

[4] Lee S.H., Choi S.H., Kim S.Y., Choi J.I., Lee J.R., Youn J.R. (2010). Degradation and Dynamic Properties of Poly(amide-co-imide)/Carbon Nanotube Composite Films. Polym. Polym. Compos..

[5] Kuan C.F., Chiang C.L., Lin S.H., Huang W.G., Hsieh W.Y., Shen M.Y. (2018). Characterization and properties of graphene nanoplatelets/XNBR nanocomposites. Polym. Polym. Compos..

[6] Anwer T., Mohammad F. (2015). Thermal stability of electrical properties and amine vapour sensitivity of in-situ prepared polyaniline/graphene nanocomposites assisted by sodium dodecyl sulfate micelles. Polym. Polym. Compos..

[7] Bian J., Lin H.L., Wang G., Zhou Q., Wang Z.J., Zhou X., Lu Y., Zhao X.W. (2016). Morphological, mechanical and thermal properties of chemically bonded graphene oxide nanocomposites with biodegradable poly (3-hydroxybutyrate) by solution intercalation. Polym. Polym. Compos..

[8] Xu B., Tanaka S.I. (1997). Formation of a new electric material: Fullerene/metal polycrystalline film. MRS Online Proc. Libr..

[9] Yu Z., Meng J., Li Y., Li Y. (2013). Efficient photocatalytic hydrogen production from water over a CuO and carbon fiber comodified TiO2 nanocomposite photocatalyst. Int. J. Hydrogen Energy.

[10] Qureshi A., Mergen A., Eroğlu M.S., Singh N.L., Güllüoğlu A. (2008). Dielectric properties of polymer composites filled with different metals. J. Macromol. Sci. Part. A.

[11] Akhavan A., Khoylou F., Ataeivarjovi E. (2017). Preparation and characterization of gamma irradiated Starch/PVA/ZnO nanocomposite films. Radiat. Phys. Chem..

[12] Rusu M., Sofian N., Ibanescu C., Rusu D. (2000). Mechanical and thermal properties of copper-powder-filled high density polyethylene composites. Polym. Polym. Compos..

[13] Lee J.S., Choi Y.J., Park H.H., Chul Pyun J. (2011). Electrochromic properties of poly (3,4-ethylenedioxythiophene) nanocomposite film containing SiO_2_ nanoparticles. J. Appl. Polym. Sci..

[14] Ou C.F., Shen P.H. (2012). Thermal and gas barrier properties of COC/SiO_2_ nanocomposites. Polym. Polym. Compos..

[15] Poisson C., Guerengomba J., Lacrampe M.F., Krawczak P., Gupta B., Miri V., Lefebvre J.M. (2008). Mechanical, optical and barrier properties of PA6/nanoclay-based single-and multilayer blown films. Polym. Polym. Compos..

[16] Zhang J., Ji Q., Shen X., Xia Y., Tan L., Wang F., Kong Q. (2012). Flame Retardancy and Non-isothermal Crystallization Behaviour of PET/TiO_2_ Nanocomposites. Polym. Polym. Compos..

[17] Ramesan M.T. (2015). Poly (Ethylene-Co-Vinyl Acetate)/Magnetite Nanocomposites: Interaction of Some Liquid Fuels, Thermal and Oil Resistance Studies. Polym. Polym. Compos..

[18] Farahmandjou M., Golabiyan N. (2019). Synthesis and characterisation of Al_2_O_3_ nanoparticles as catalyst prepared by polymer co-precipitation method. Mater. Eng. Res..

[19] Chen Y.H., Yao X.Y., Pan Z.J., Gu Q. (2011). Preparation and isothermal crystallization behavior of poly (lactic acid)/graphene nanocomposites. Adv. Mater. Res..

[20] Zhang Y., Huang L.-j., Wang Y.-x., Tang J.-g., Wang Y., Cheng M.-m., Du Y.-c., Yang K., Kipper M.J., Hedayati M. (2019). The preparation and study of ethylene glycol-modified graphene oxide membranes for water purification. Polymers.

[21] Ahmed M., Kishi N., Sugita R., Fukaya A., Khatri I., Liang J., Jimbo T. (2013). Graphene synthesis by thermal chemical vapor deposition using solid precursor. J. Mater. Sci. Mater. Electron..

[22] Fan Y.C., Wang L.J., Jiang W. (2018). Graphene based oxide ceramic composites with high mechanical and functional performance: From preparation to property. J. Inorg. Mater..

[23] Al-Tikrity E.T.B., Waheed I.F., Ali S.M. (2019). Study of electrical properties of a reduced graphene-oxadiazole-2-thiol (rGS) PVA polymer composite. Polym. Polym. Compos..

[24] Alizadeh T., Ahmadian F. (2015). Thiourea-treated graphene aerogel as a highly selective gas sensor for sensing of trace level of ammonia. Anal. Chim. Acta.

[25] Mousavi H. (2010). Doped graphene as a superconductor. Phys. Lett. A.

[26] Tan L., Zhang Y.C., Wang B., Luo H.M., Feng H.X. (2014). Reduced Graphene Oxide as a Metal-Free Carbocatalyst for Polymerization of 1-Naphthylamine. ChemPlusChem.

[27] Song E., Han W., Li C., Cheng D., Li L., Liu L., Zhu G., Song Y., Tan W. (2014). Hyaluronic acid-decorated graphene oxide nanohybrids as nanocarriers for targeted and pH-responsive anticancer drug delivery. ACS Appl. Mater. Interfaces.

[28] Zamharir M.J., Asl M.S., Kakroudi M.G., Vafa N.P., Zamharir M.J. (2015). Significance of hot pressing parameters and reinforcement size on sinterability and mechanical properties of ZrB2–25 vol% SiC UHTCs. Ceram. Int..

[29] Tsou C.H., Yao W.H., Hung W.S., Suen M.C., De Guzman M.R., Chen J., Tsou C.Y., Wang R.Y., Chen J.C., Wu C.S. (2018). Innovative plasma process of grafting methyl diallyl ammonium salt onto polypropylene to impart antibacterial and hydrophilic surface properties. Ind. Eng. Chem. Res..

[30] Ahmad J., Burduhos-Nergis D.D., Arbili M.M., Alogla S.M., Majdi A., Deifalla A.F. (2022). A review on failure modes and cracking behaviors of polypropylene fibers reinforced concrete. Buildings.

[31] De Albuquerque T.L., Júnior J.E.M., de Queiroz L.P., Ricardo A.D.S., Rocha M.V.P. (2021). Polylactic acid production from biotechnological routes: A review. Int. J. Biol. Macromol..

[32] Ilyas R.A., Zuhri M.Y.M., Aisyah H.A., Asyraf M.R.M., Hassan S.A., Zainudin E.S., Sari N.H. (2022). Natural fiber-reinforced polylactic acid, polylactic acid blends and their composites for advanced applications. Polymers.

[33] Tsou C.Y., Wu C.L., Tsou C.H., Chiu S.H., Suen M.C., Hung W.S. (2015). Biodegradable composition of poly (lactic acid) from renewable wood flour. Polym. Sci. Ser. B.

[34] Tsou C.H., Kao B.J., Suen M.C., Yang M.C., Wu T.Y., Tsou C.Y., Lai J.Y. (2014). Crystallisation behaviour and biocompatibility of poly (butylene succinate)/poly (lactic acid) composites. Mater. Res. Innov..

[35] Mittal A., Garg S., Premi A., Giri A.S. (2021). Synthesis of polyvinyl alcohol/modified starch-based biodegradable nanocomposite films reinforced with starch nanocrystals for packaging applications. Polym. Polym. Compos..

[36] Al-Muntaser A.A., Pashameah R.A., Sharma K., Alzahrani E., Farea M.O., Morsi M.A. (2022). α-MoO_3_ nanobelts/CMC-PVA nanocomposites: Hybrid materials for optoelectronic and dielectric applications. J. Polym. Res..

[37] Afzal H.M., Iqbal MItu S.S., Al-Harthi M.A. (2018). Microwave radiations effect on electrical and mechanical properties of poly (vinyl alcohol) and PVA/graphene nanocomposites. Surf. Interfaces.

[38] Chen W., Wu K., Liu Q., Lu M. (2020). Functionalization of graphite via Diels-Alder reaction to fabricate poly (vinyl alcohol) composite with enhanced thermal conductivity. Polymer.

[39] Mahendia S., Chahal R.P., Munjal N., Kumar S. (2014). Graphene-Polyvinyl Alcohol (PVA) Composites with Enhanced Conductivity: A Comparative Study of Various Synthesis Approaches. Adv. Electrochem..

[40] Liu Y., Wu K., Luo F., Lu M. (2019). Significantly enhanced thermal conductivity in polyvinyl alcohol composites enabled by dopamine modified graphene nanoplatelets. Compos. Part. A Appl. Sci. Manuf..

[41] Morimune S., Nishino T., Goto T. (2012). Poly (vinyl alcohol)/graphene oxide nanocomposites prepared by a simple eco-process. Polym. J..

[42] Li K.M., Dong H., Lin H., Zhang J.K. (2017). Study on the thermal conductivity of graphene oxide/polyvinyl alcohol composites. J. Eng. Therm. Energy Power.

[43] Chen Z.C., Chang T.L., Pan T.C., Chiang D., Tseng S.F. (2018). A facile approach to fabrication and characterization of conductive conjugated polyvinyl alcohol/graphene composite nanofibers. Mater. Lett..

[44] Peng Y.Y., Hsieh T.E., Hsu C.H. (2005). White-light emitting ZnO–SiO_2_ nanocomposite thin films prepared by the target-attached sputtering method. Nanotechnology.

[45] Tamulevičius T., Peckus D., Tamulevičiene A., Vasiliauskas A., Čiegis A., Meškinis Š., Tamulevičius S. Dynamic optical properties of amorphous diamond-like carbon nanocomposite films doped with Cu and Ag nanoparticles. Proceedings of the Plasmonics: Metallic Nanostructures and Their Optical Properties XII.

[46] Zahid M., Ali S., Saleem S., Salman M., Khan M. (2020). Carbon nanoparticles/polyvinyl alcohol composites with enhanced optical, thermal, mechanical, and flame-retardant properties. J. Appl. Polym. Sci..

[47] Dragoman M., Modreanu M., Povey I.M., Dinescu A., Dragoman D. (2019). Reconfigurable horizontal–vertical carrier transport in graphene/HfZrO field-effect transistors. Nanotechnology.

[48] Kim S., Shimazu J., Fukaminato T., Ogata T., Kurihara S. (2017). Thermal conductivity of graphene oxide-enhanced polyvinyl alcohol composites depending on molecular interaction. Polymer.

[49] Park G.T., Chang J.H. (2019). Comparison of Properties of PVA Nanocomposites Containing Reduced Graphene Oxide and Functionalized Graphene. Polymers.

[50] Wang J., Wang X., Xu C., Zhang M., Shang X. (2011). Preparation of graphene/poly (vinyl alcohol) nanocomposites with enhanced mechanical properties and water resistance. Polym. Int..

[51] Zhao X., Zhang Q., Chen D.J., Lu P. (2010). Enhanced Mechanical Properties of Graphene-Based Poly(vinyl alcohol) Composites. Macromolecules.

[52] Tao C.A., Zhang H., Huang J., Zou X., Zhu H., Wang J. (2017). Reduction versus cross-linking: How to improve the tensile strength of graphene oxide/polyvinyl alcohol composite film. Mater. Res. Express.

[53] Park J.H., Kim I.K., Choi J.Y., Karim M.R., Cheong I.W., Oh W., Yeum J.H. (2011). Electrospinning Fabrication of polyvinyl alcohol)/waterborne polyurethane/silver composite nanofibre mats in aqueous solution for anti-bacterial exploits. Polym. Polym. Compos..

[54] Hu H., Chen G. (2010). Electrochemically modified graphite nanosheets and their nanocomposite films with poly (vinyl alcohol). Polym. Compos..

[55] Pope C.G. (1997). X-ray diffraction and the Bragg equation. J. Chem. Educ..

[56] Jung S.Y., Paik K.W. Effects of alignment of graphene flakes on water permeability of graphene-epoxy composite film. Proceedings of the 2014 IEEE 64th Electronic Components and Technology Conference (ECTC).

[57] Mehta J., Vinayak P., Tuteja S.K., Chhabra V.A., Bhardwaj N., Paul A.K., Deep A. (2016). Graphene modified screen printed immunosensor for highly sensitive detection of parathion. Biosens. Bioelectron..

[58] Pfaffeneder-Kmen M., Casas I.F., Naghilou A., Trettenhahn G., Kautek W. (2017). A Multivariate Curve Resolution evaluation of an in-situ ATR-FTIR spectroscopy investigation of the electrochemical reduction of graphene oxide. Electrochim. Acta.

[59] Mawhinney D.B., Yates J.T. (2001). FTIR study of the oxidation of amorphous carbon by ozone at 300 K—Direct COOH formation. Carbon.

[60] Vineeth S.K., Gadhave R.V., Gadekar P.T. (2023). Polyvinyl alcohol–cellulose blend wood adhesive modified by citric acid and its effect on physical, thermal, mechanical and performance properties. Polym. Bull..

[61] Bian Y., Colin X., Aressy M. (2020). Thermal aging of high tenacity polyvinyl alcohol yarns. Polym. Degrad. Stab..

[62] Greczynski G., Hultman L. (2021). The same chemical state of carbon gives rise to two peaks in X-ray photoelectron spectroscopy. Sci. Rep..

[63] Isaacs M.A., Davies-Jones J., Davies P.R., Guan S., Lee R., Morgan D.J., Palgrave R. (2021). Advanced XPS characterization: XPS-based multi-technique analyses for comprehensive understanding of functional materials. Mater. Chem. Front..

[64] Cortez-Lemus N.A., Salgado-Rodríguez R., Licea-Claveríe A. (2010). Preparation of α, ω-telechelic hexyl acrylate polymers with —OH, —COOH, and —NH_2_ functional groups by RAFT. J. Polym. Sci. Part. A Polym. Chem..

[65] Mirabedini S.M., Zareanshahraki F., Mannari V. (2020). Enhancing thermoplastic road-marking paints performance using sustainable rosin ester. Prog. Org. Coat..

[66] Zhang X., Zhou J., Zheng Y., Wei H., Su Z. (2021). Graphene-based hybrid aerogels for energy and environmental applications. Chem. Eng. J..

[67] Liu Y., Liu Y., Jiang H., Xu L., Cheng Y., Wang P.G., Wang F. (2014). Preparation, antiangiogenic and antitumoral activities of the chemically sulfated glucan from Phellinus ribis. Carbohydr. Polym..

[68] Vitale A., Merlo S., Rizza G., Melilli G., Sangermano M. (2014). UV Curing of Perfluoropolyether Oligomers Containing Graphene Nanosheets to Enhance Water-Vapor Barrier Properties. Macromol. Chem. Phys..

[69] Yoo B.M., Shin H.J., Yoon H.W., Park H.B. (2014). Graphene and graphene oxide and their uses in barrier polymers. J. Appl. Polym. Sci..

[70] Tsou C.H., Ge F.F., Lin L., Yuan S., De Guzman M.R., Potiyaraj P. (2023). Barrier and Biodegradable Properties of Poly (butylene adipate-co-terephthalate) Reinforced with ZnO-Decorated Graphene Rendering it Antibacterial. ACS Appl. Polym. Mater..

[71] Bian Q., Tian H., Wang Y., Liu Q., Ge X., Rajulu A.V. (2015). Effect of graphene oxide on the structure and properties of poly (vinyl alcohol) composite films. Polym. Sci. Ser. A.

[72] Liu D., Bian Q., Li Y., Wang Y., Tian H. (2016). Effect of oxidation degrees of graphene oxide on the structure and properties of poly (vinyl alcohol) composite films. Compos. Sci. Technol..

[73] Liu Q., Ge X., Tian H. (2016). Effect of copper sulfate pentahydrate on the structure and properties of poly (vinyl alcohol)/graphene oxide composite films. J. Appl. Polym. Sci..

[74] Wang X., Li Y., Chen Y., Guo L., Wang Q., Xu C. (2018). Preparation and properties of functional graphene/polyvinyl alcohol composite films. J. For. Eng..

[75] Genorio B., Harrison K.L., Connell J.G., Dražić G., Zavadil K.R., Markovic N.M., Strmcnik D. (2019). Tuning the selectivity and activity of electrochemical interfaces with defective graphene oxide and reduced graphene oxide. ACS Appl. Mater. Interfaces.

[76] Lim J.V., Bee S.T., Tin Sin L., Ratnam C.T., Abdul Hamid Z.A. (2021). A review on the synthesis, properties, and utilities of functionalized carbon nanoparticles for polymer nanocomposites. Polymers.

[77] Dorigato A., Dzenis Y., Pegoretti A. (2013). Filler aggregation as a reinforcement mechanism in polymer nanocomposites. Mech. Mater..

[78] Wen Y.H., Tsou C.H., de Guzman M.R., Huang D., Yu Y.Q., Gao C., Wang Z.H. (2022). Antibacterial nanocomposite films of poly (vinyl alcohol) modified with zinc oxide-doped multiwalled carbon nanotubes as food packaging. Polym. Bull..

[79] Salavagione H.J., Martínez G., Gómez R., Segura J.L. (2010). Synthesis of water-soluble perylenediimide-functionalized polymer through esterification with poly (vinyl alcohol). J. Polym. Sci. Part. A Polym. Chem..

[80] Kapourani A., Chachlioutaki K., Andriotis E.G., Fatouros D.G., Barmpalexis P. (2023). Evaluating PAA/PVA thermal crosslinking process during the preparation of in-situ high-drug loading amorphous solid dispersions. J. Drug Deliv. Sci. Technol..

[81] Van der Schueren B., El Marouazi H., Mohanty A., Lévêque P., Sutter C., Romero T., Janowska I. (2020). Polyvinyl alcohol-few layer graphene composite films prepared from aqueous colloids. Investigations of mechanical, conductive and gas barrier properties. Nanomaterials.

[82] Hwang E.H., Sarma S.D. (2009). Screening-induced temperature-dependent transport in two-dimensional graphene. Phys. Rev. B.

[83] Li J., Wang G., Zhu H., Zhang M., Zheng X., Di Z., Wang X. (2014). Antibacterial activity of large-area monolayer graphene film manipulated by charge transfer. Sci. Rep..

[84] Bao Y., Wei C., Su E., Zhang Q., Bai Y. (2013). Study on the synthesis and antibacterial properties of silver-chitosan composite. Polym. Polym. Compos..

[85] Mallakpour S., Abdolmaleki A., Khalesi Z. (2018). Fabrication and physicochemical features study of crosslinked PVA/FGO nanocomposite films. Polym. Bull..

[86] Wu H., Drzal L.T. (2012). Graphene nanoplatelet paper as a light-weight composite with excellent electrical and thermal conductivity and good gas barrier properties. Carbon.

[87] Yao Y.L., De Guzman M.R., Duan H., Gao C., Lin X., Wen Y.H., Tsou C.H. (2020). Infusing high-density polyethylene with graphene-zinc oxide to produce antibacterial nanocomposites with improved properties. Chin. J. Polym. Sci..

[88] Ma Z.L., Tsou C.H., Yao Y.L., De Guzman M.R., Wu C.S., Gao C., Yang T., Chen Z.J., Zeng R., Li Y. (2021). Thermal properties and barrier performance of antibacterial high-density polyethylene reinforced with carboxyl graphene-grafted modified high-density polyethylene. Ind. Eng. Chem. Res..

[89] Liu S., Zeng T.H., Hofmann M., Burcombe E., Wei J., Jiang R., Chen Y. (2011). Antibacterial activity of graphite, graphite oxide, graphene oxide, and reduced graphene oxide: Membrane and oxidative stress. ACS Nano.

[90] Akhavan O., Ghaderi E. (2010). Enhancement of antibacterial properties of graphene sheets decorated with silver nanoparticles. J. Mater. Chem..

