# Supplementary material for: Enhancing Polyvinyl Alcohol Nanocomposites with Carboxy-Functionalized Graphene: An In-Depth Analysis of Mechanical, Barrier, Electrical, Antibacterial, and Chemical Properties
Source: Polymers (Basel). 2024 Apr 11;16(8):1070. doi: 10.3390/polym16081070 (PMC11054367; doi:10.3390/polym16081070)
Supplement: Supplementary file 1 [file polymers-16-01070-s001.zip › polymers-2858030-supplementary.pdf]

Supplementary materials

Table S1. Mechanical properties of PVA and nanocomposite 3%MG/PVA.

Sample	Process	Tensile strength (MPa)	Yield strength (MPa)	Elongation at break (%)
Process 1	U1+U2	43.1±1.9	21.8±1.1	31.5±6.2
Process 2	U2+U3	45.6±2.5	22.3 ±2.4	35.9±5.8
Process 3	U1+U3	34.7±3.6	18.6±2.6	27.4±4.4
Process 4	U1+U2+U3	46.8±2.3	23.5±3.1	35.7±5.6
